# Sustainable malaria control: transdisciplinary approaches for translational applications

**DOI:** 10.1186/1475-2875-11-431

**Published:** 2012-12-26

**Authors:** Lyn-Marie Birkholtz, Riana Bornman, Walter Focke, Clifford Mutero, Christiaan de Jager

**Affiliations:** 1 Department of Biochemistry, University of Pretoria, Private Bag x20, Pretoria, Gauteng, South Africa, 0028; 2 University of Pretoria Centre for Sustainable Malaria Control (UP CSMC), University of Pretoria, Private Bag x323, Pretoria, Gauteng, South Africa, 0001; 3 Department of Chemical Engineering, University of Pretoria, Private Bag x20, Pretoria, Gauteng, South Africa, 0028; 4 International Centre of Insect Physiology and Ecology, P.O. Box 30772, Nairobi, Kenya, 00100

**Keywords:** *Plasmodium*, *Anopheles*, DDT, Vector control, Anti-malarial drugs

## Abstract

With the adoption of the Global Malaria Action Plan, several countries are moving from malaria control towards elimination and eradication. However, the sustainability of some of the approaches taken may be questionable. Here, an overview of malaria control and elimination strategies is provided and the sustainability of each in context of vector- and parasite control is assessed. From this, it can be concluded that transdisciplinary approaches are essential for sustained malaria control and elimination in malaria-endemic communities.

## Background

Malaria is still the most important parasitic disease in humans, caused by *Plasmodium spp.* parasites that are transmitted through the bite of female *Anopheles* mosquitoes. Despite recent massive global efforts in malaria control, about 40% of the world’s population in 108 countries still live under the constant risk of malaria infection [1]. The World Health Organization estimates that ~225 million clinical malaria episodes occur annually and more than 80% of malaria-associated deaths in the world occur in sub-Saharan Africa [1]. This disease, therefore, rates as one of the major health, socio-economic and developmental challenges facing many of the world’s poorest countries. As a disease of poverty, it forms a major impediment to economic development on the African continent, as it is responsible for the loss of ~US$12 billion of gross domestic product annually. The Millennium Development Goals of the United Nations Development Programme underscore the importance of combating malaria in Target 6C: “to have halted and begun to reverse the incidence of other major diseases by 2015”. Malaria is both one of the major disease role players and, in the African context, one of the three major causes of childhood mortality. Thus, clear motivation exists supporting global control of the disease and sustainably eliminating it in areas where transmission has been successfully interrupted. Ultimately, the vision of global eradication should drive all current initiatives, investments and decision-making.

A number of international initiatives have been established to coordinate efforts for its control, elimination and, ultimately, eradication. Towards this end, the Global Malaria Action Plan was adopted in 2007, supported by the WHO/Roll Back Malaria Partnership [2] and global funding mechanisms including the Global Fund and the Bill & Melinda Gates Foundation. Currently, 81 countries are enforcing malaria control while 25 are in pre-elimination and prevention-of-reintroduction phases, with four countries recently being declared malaria free [1]. Under the WHO definition, “malaria elimination” refers to a situation where there is zero local transmission, whereas “eradication” is defined as the permanent zero worldwide incidence of malaria infection [1].

### Factors impeding elimination and eradication: sustainability

Global efforts to eradicate infectious diseases have been successful for smallpox, but in reality, this was due to the simplicity of this organism with visible pathogenesis in patients, which allows very easy monitoring of infectious cases and application of a useful, point-of-care vaccine [3]. However, other major infectious diseases such as poliomyelitis have not been completely eradicated, with several African countries showing an alarming re-introduction of the disease.

**Figure 1:**
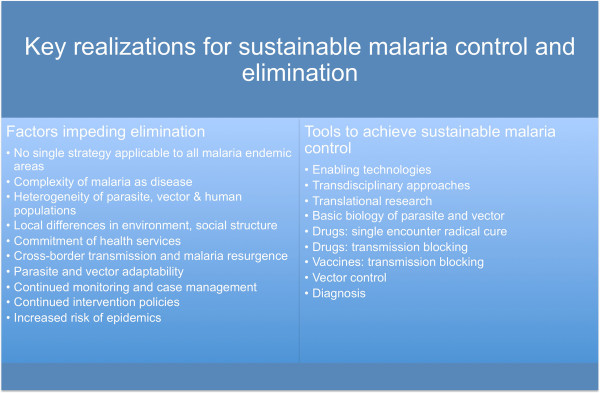
Key realizations impeding and, in contrast, enabling sustainability in malaria control and elimination.

Therefore, if the malaria community is to achieve elimination and eradication, the key realization is that this has to be approached with the long-term goal of sustainability (Figure 1). Several times during our history global efforts have been initiated to control malaria, all with less than satisfactory outcomes (e.g., Global Malaria Eradication Programme, 1955–1969), even though malaria elimination has been achieved in several areas of the world. One of the major realizations from previous malaria-elimination attempts was that, compared to smallpox and poliomyelitis, no single strategy will be applicable for controlling and eliminating malaria. However, to succeed with the current global malaria eradication programme, it has to be understood that, unlike other infectious diseases including smallpox, human immunodeficiency virus infections and tuberculosis, malaria is not caused by a single biological entity. Rather, the disease is the result of a highly complex interplay between three biological systems – including >30 species of *Anopheles* mosquitoes acting as vectors for the parasite, as well as five species of Plasmodia that infect humans (*Plasmodium falciparum; Plasmodium vivax, Plasmodium ovale; Plasmodium malariae; Plasmodium knowlesi*), each with its own complex lifecycle, environment, habits and pathogenesis profiles. Additionally, the current global environment conspires in the transmission of malaria with, for instance, travellers’ malaria repeatedly reintroducing the disease and global climatic changes forecasting a significant increase in malaria cases. In this context, countries aiming at malaria elimination but which are bordered by countries still experiencing malaria transmission will always be in a situation where their elimination efforts will require strict monitoring and sustained malaria control (Figure 1). Lastly, in countries aiming at zero local transmission, or achieving pre-elimination stages, there will still be a substantial requirement in support of sustained malaria control such that: 1) parasite and vector adaptability is pre-empted; 2) local differences in environment and social structures are taken into consideration; 3) malaria-related health services are maintained in endemic regions; 4) malaria monitoring and case management are continued; 5) continued maintenance of intervention policies will remain imperative; and 6) increased risk of epidemics are monitored (Figure 1).

### Sustainable malaria control: what is needed?

With the classic definition of sustainability implying the ability to maintain an effort at a certain level or rate, sustainable malaria control should, therefore, be seen as long-term effort(s) focused on decreasing malaria-associated fatalities through integrated, creative transdisciplinary approaches that will ultimately contribute to malaria elimination and eradication. Transdisciplinarity in this context requires the combination of basic biomedical sciences and public health efforts and the inclusion of the management of malaria in education and policy making, thereby creating a cross-boundary discipline with a singular focus area. Not only should this lead to translational research with the transfer of knowledge from ‘bench-to-bed’ but, also, the knowledge from health aspects of malaria control mechanisms should guide and focus research efforts in the basic sciences. The major technical advances that were not available to scientists in the 1950s and 1960s during the previous global malaria-eradication attempts should now be exploited, research from the biomedical field should essentially be applied to the field and lastly, and most importantly for sustainable malaria control, major efforts have to be invested in to obtain a clearer understanding of the biology of both the insect vector as well as the parasite itself. In addition, human health aspects of any intervention strategy needs close considerations. Sustained malaria control can be achieved by the use of various physical and biological vector and parasite control strategies aimed at controlling malaria transmission (including drugs, vaccines and innovative vector control strategies), as well as effective tools for malaria diagnosis as well as monitoring and surveillance. The following sections will discuss various aspects of vector and parasite control strategies for controlling malaria transmission, but will not dwell on vaccines or diagnostics, as these are extensively covered elsewhere (e.g. [4,5]).

### Vector control strategies and sustainable malaria control

Vector control is among the key strategies that have significantly contributed to a reduction in malaria illness and deaths in a number of African countries during the last decade [6-9]. The goal of malaria vector control is to reduce vector capacity to below the threshold required to maintain the malaria infection rate. The success so far achieved in malaria vector control has primarily been ascribed to the scaling up of two interventions [6,7]: the use of long-lasting insecticide-treated nets (LLINs or ITNs [10,11]) and indoor residual spraying of long-lasting insecticides (IRS [12]). The WHO’s World Malaria Report (2010) states that by 2015, every person at risk for malaria must have either an ITN or access to IRS [1]. Besides the above, malaria vectors can also be controlled through larval source management using larvicides, i.e. insecticides that specifically target the larval life stage of the mosquito, and biological agents including bacterial larvicides, fungi and larvivorous fish (Table 1[13,14]). Larval source management has been successfully applied as the primary mosquito control method in several US districts, in Canada, Brazil, Singapore and throughout Europe and could be successfully applied in the African context [15]. Additionally, both larval and adult vector populations can be controlled with environmental management [16-19], including permanent infrastructural changes of a capital-intensive nature and creating temporary unfavourable conditions for vector breeding. Without evidence of efficacy of *en masse* employment of these alternative control strategies, it is at this stage unclear if these will ultimately be sustainable as vector control strategies.

**Table 1:** Potential sustainability of vector elimination strategies in malaria control, elimination and eradication programmes

**Subgroup**	**Advantages**	**Disadvantages**	**Sustainability**
**Aerial spraying**[20]	Cost-effective and simple to implement	Only effective for controlling exophilic mosquitoes	It may fail as it does not target indoor feeding mosquitoes, which are responsible for the bulk of malaria transmission
**Larviciding**[21,22]	Can eliminate sources of mosquitoes	There is need to develop a cheap and effective larvicide. Insect growth regulators are expensive and limited in availability.	*An. gambiae* was eliminated in Brazil using arsenic larviciding
**Environmental control**[16]	Can lead to vector elimination	Environmental modification is expensive and may be possible only in more advanced economies	Potential to bring about vector elimination
**Biological control**[16,23]	Effective if implemented correctly	Expensive and technically challenging for poorer countries	Potential to bring about vector elimination

Both primary methods of vector control, LLINs and IRS, rely on the use of long-lasting insecticides recommended for public health use by the WHO (Table 2). Currently, the insecticides most commonly used for LLINs belong to the pyrethroid class while those for IRS are either pyrethroids or members of three other classes: carbamates, organophosphates, and organochlorine insecticides such as dichlorodiphenyltrichloroethane (DDT). Unfortunately, the recent emergence of vector resistance to the most commonly used insecticides undermines the sustainability of recent gains in malaria control. According to a recent WHO report, insecticide resistance has been identified in 64 countries, most of them in sub-Saharan Africa, but also in India [24]. A number of countries continue using DDT as a viable vector control tool, especially in areas of pyrethroid resistance [25,26]. However, the continued dependence on the above-mentioned insecticides for vector control is eminently unsustainable in view of insecticide resistance and potential health and environmental risks.

**Table 2:** Potential sustainability of transmission blocking strategies in malaria control, elimination and eradication programmes

	**Subgroup**	**Advantages**	**Disadvantages**	**Sustainability**
**Prevention of mosquito bites**	**ITNs/LLINs**[1,10,11]	Cheap and easy to implement	Only offers protection during sleeping time	Mosquitoes can still transmit malaria before sleeping time
	**Repellents**[27]	Effective in preventing bites	Short residual efficacy, strong smell, irritating to the skin	Does not reduce vector populations; mosquitoes will simply migrate to areas where repellents are not in use
	**Attractants**[28]	Safe for humans and environment, cheap	Chemicals that attract have not been fully isolated	Very promising technique
	**House design**[29]	Very effective and cheap	Closing up eaves increases indoor temperatures	Does not reduce vector populations, but worked well for Europe and North America
**Killing of mosquitoes after they have bitten**	**IRS**[1,12]	Breaks transmission cycle	Too much reliance on DDT; dusting of sprayed insecticides a problem, labour-intensive	Residual efficacy limited to at most one season
	**ITWL**[30,31]	Similar to IRS but eliminates dusting and short residual efficacy of insecticides	User acceptability may be a challenge	Emerging polymer technology will eliminate the need to spray chemicals
	**IRS/ITWL with natural insecticides**[32,33]	Low mammalian toxicity	Short residual efficacy	Pyrethrin is the most effective insecticide

### The effects of vector control strategies on human health

A growing body of evidence suggests that exposure to DDT and its breakdown product DDE may be associated with adverse health outcomes in humans [34,35]. The following health conditions showed significant associations with DDT or DDE exposure [36]: menstrual cycle alterations [37,38]; foetal loss in women in countries with recent usage of DDT [39,40]; reduced gestational age and increased rates of preterm birth [41]; breast cancer pre-pubertal exposure (prior to age 14, not later) [42]; testicular cancer prospective exposure measure-provided evidence [43]; liver cancer [44]; reduced childhood or pubertal growth after prenatal or early life exposures to DDE [45]; DNA damage and apoptosis [46] and impaired neurodevelopment up to age four [47,48]. Eskenazi *et al.*[35] described growing evidence that DDT exposures may be associated with adverse health outcomes for breast cancer, diabetes, decreased semen quality, spontaneous abortion, and impaired neurodevelopment in children. Higher rates of urogenital malformations in newborn boys of mothers who live in DDT-treated areas have also been reported, although a quantitative human health risk assessment could not be performed [49].

However, for several other health conditions, the effects of DDT or DDE are inconclusive or the data insufficient to draw clear conclusions. These include: exposure to DDT during lactation; influence of exposure on age or menarche or pubertal stages; time to pregnancy as an indicator of fertility or fecundity; thyroid hormones; lung/pancreatic/endometrial or prostate cancers; non-Hodgkin’s lympohoma; diabetogenic effects; immunotoxicity; cryptorchidims; and anogentical distance or hypospadias. Additionally, no significant associations with DDT or DDE were demonstrated for foetal growth restriction, cancer incidence or mortality, including breast cancer at the time of diagnosis or during adulthood [36]. Owing to these inconclusive and conflicting datasets, DDT use is approved by the WHO.

Pyrethroid insecticides are often used as an alternative to DDT in IRS programmes, especially deltamethrin [26]. However, animal studies suggest that prenatal exposure to pyrethroids may also adversely affect neurodevelopment and this raises concern for people living in malarial areas where IRS is conducted with both chemicals [36]. Additionally, pyrethroids are often used for agricultural purposes and domestic pest control, which may further increase total pyrethroid exposure and increase instances of pyrethroid resistance developing and impeding sustainable use of this insecticide.

In general, there are many instances where the data on health effects of insecticide usage are inconclusive and consequently adverse health effects in the currently exposed populations or their offspring (via epigenetic mechanisms) cannot be ruled out. Information on exposures to children living in IRS communities is virtually non-existent and research has to focus on both foetal and neonatal exposures to determine the long-term safe use of these chemicals related to IRS. Filling those gaps in high-quality data on risks to humans challenges scientists to use opportunities in currently exposed populations to draw scientifically justifiable conclusions. Since there is a growing societal awareness about health and the environment, uncertainties about current strategies will eventually only limit long-term sustainability.

### Sustainable and alternative vector control strategies

It is estimated that existing malaria vector control interventions can only reduce the annual inoculation rate by an order of magnitude [50]. This suggests that the interventions currently recommended by the WHO may not be sufficient to achieve malaria elimination in Africa. There is therefore a need for effective vector control interventions that can deal a “knockout blow” to malaria vectors in a sustainable manner.

### Integrated vector management

Integrated vector management (IVM) could be seen as a key strategy for sustainable malaria vector control and as a rational decision about the optimal use of resources for vector control [51-53]. IVM aims to improve the efficacy, cost effectiveness, ecological soundness and sustainability of vector control to prevent or interrupt disease transmission [54] by using a range of control options that can respond better to the natural variation and heterogeneity of vector populations [55]. The key elements of IVM can be summarized as: 1) effective targeting of different vector species and developmental stages of vectors in a given geographical area; 2) combining of chemical- and non-chemical methods in order to arrest the development of insecticide resistance in vector populations and minimize the negative health and environmental impacts of chemicals [51,54]; 3) inter-sectoral collaborations between health and agricultural sectors, for example, for effective implementation of IVM; and 4) development of the requisite human resources, including the training of a cadre of IVM practitioners in entomological and program management skills.

Behavioural and ecological variations in mosquito vectors are readily evident in relation to the day-time resting behaviour of fed adult mosquitoes of the *Anopheles gambiae* species complex (= *Anopheles gambiae sensu lato* [s.l.]) and *Anopheles funestus*[56] found in Africa. *Anopheles gambiae s.s* and *An. funestus* rest almost exclusively indoors under natural conditions and feed exclusively on human hosts. In contrast, *Anopheles arabiensis* normally rests either indoors or outdoors in a variety of micro-habitats such as ground pits and feeds either indoors or outdoors on human hosts or domestic animals [57]. It is therefore obvious that interventions aimed at the various species need to take different behavioural variability into account to be fully effective. On the one hand, the control of *An. gambiae s.s* and *An. funestus* can be achieved by largely targeting the adult mosquitoes indoors, since this is where they normally feed and rest before leaving houses in search of oviposition sites. However, continuous use of insecticides indoors to target *An. gambiae s.s* and *An. funestus* is now known to change the behaviour of certain vector populations such that these vectors end up feeding and resting more frequently outdoors than indoors or start to bite early in the evenings before people sleep under the protection of bed nets [58].

IVM additionally underscores the combination of chemical and non-chemical methods in order to arrest the development of insecticide resistance in vector populations and minimize the negative health and environmental impacts of chemicals [51,54]. Non-chemical methods including the use of bio-larvicides (Table 1) and environmental management may prove as successful as chemical means and be applicable to sustainable programmes. Environmental management for both vector and larvae control includes closely related strategies namely environmental modification (e.g. infrastructural changes) and environmental manipulation (e g achieving unfavourable conditions for vector breeding including ditch filling, stream flushing etc.). IVM contributes towards sustainable malaria control by mitigating and managing insecticide resistance through ensuring no over-reliance on a particular insecticide for a prolonged period of time or rotating different classes of insecticides. Environmental management of potential mosquito breeding habitats can also serve as a practical alternative, or a complementary intervention for eliminating the malaria vector problem, in areas where LLINs and IRS have been rendered ineffective by resistance.

Collaboration between the health sector and other sectors is an essential element of implementation of IVM, since activities and policies outside of the health sector can have important implications for malaria control. For example, conflicting agendas may exist between government agencies where a department of health may advocate the use of insecticides but a department of environmental affairs will spearhead national implementation plans for phasing out that exact chemical. Independent evidence-based research is, therefore, imperative for providing information related to the potential benefits and risks of controlling malaria. Moreover, the use of agricultural pesticides to protect crops from pest attack is known to lead to insecticide resistance in vectors, thus rendering interventions such as IRS and LLINs ineffective in the areas where such a problem exists [59,60]. By promoting cross-sectoral collaboration between the ministries of health and agriculture, it would be possible to minimize agriculture-associated malaria risks.

Programmatic control of malaria vectors requires that policy decisions regarding the vector control strategy, as well as the specific interventions, are made on the basis of a sound understanding of the behaviour, ecology and population dynamics of the local vector species [61]. The relevant basic and operational research therefore needs to be conducted in order to effectively target the specific vector. Such research should also facilitate the mapping of the spatial and seasonal variation in malaria-transmission risks in different geographic locations. Systematic entomological surveillance is also crucial for monitoring and evaluating the success or limitations of the different interventions being used. Research is, therefore, a key element of IVM as the means for generating the evidence base needed to guide vector control interventions within an IVM context in certain African countries.

### Prevention of mosquito bites and killing mosquitoes after they have bitten

Interventions falling in these two categories and preventing transmission from vector to host are presented in Table 2. Mosquito control by suitable house designs, that prevent mosquitoes from entering, was extensively used before the advent of DDT and pyrethroids and apparently proved to be effective [29]. Mosquito control in Europe and North America was achieved largely through suitable house design, which can significantly reduce the number of mosquitoes feeding indoors by up to 80% [29]. Additionally, successful attempts have been made to divert mosquitoes away from humans, using attractants and repellents. The attractants seem to work by inhibiting CO_2_ receptors or continuously activating CO_2_ sensors, or by simulating the smell of CO_2_, thus curtailing the ability of a mosquito to locate a victim using CO_2_ sensory neurons [28]. The use of repellents has also been shown to be effective in preventing bites from mosquitoes, of which DEET (*N,N*-diethyl-m-toluamide) is currently the most effective and most widely used repellent. DEET repels by blocking the lactic acid receptors in mosquitoes, resulting in the insect losing the trail leading to the host [27].

Larviciding, environmental management, genetic manipulation, attractants and ITWL all offer promising vector control techniques for the future. However, larviciding requires expensive applications; environmental management would require government commitment to infrastructural management; and genetic manipulation would be expensive over a sustained period of implementation and also technically challenging for poor African countries. Alternatively, durable insecticide-treated wall linings (ITWLs), a recent product concept that combines aspects of LLINs and IRS, promise long-lasting residual efficacy, negating the need for repeated annual wall treatments as currently occurs with IRS. In combination with newly identified suitable insecticide alternatives using slow-release formulations [62,63], ITWL can be used as a substitute for IRS, since it allows protective containment of the insecticide in a polymer, resulting in a longer residual efficacy. Additionally, natural insecticides including permethrin or pyrethrin and essential oils such as thymoquinone, nootkatone and carvacol [32] should be explored if their residual period of use could be increased [33].

### Sustainable parasite control

Although there are alternatives to insecticides for vector control, parasite control is still fully reliant on anti-malarial drugs, both for prophylactic use and for treatment of the disease. The addition of a vaccine to the anti-malarial weaponry will enable long-term protection that cannot be achieved by chemical interventions, but it is unlikely that either a vaccine on its own or control of the parasite on its own will ever be effective for sustainable malaria control, which necessitates vigilant coordination of the available control strategies. This is particularly true if malaria eradication is to be achieved by reducing parasite numbers to prevent parasite transmission. However, malaria control is fragile and current control programmes are threatened by various factors, the most alarming of which is the rapid development of drug- and insecticide-resistant forms of the malaria parasite and the mosquito vector, respectively. This is particularly significant for *P. falciparum* parasites, which have to various degrees developed resistance against all currently used anti-malarials.

**Figure 2:**
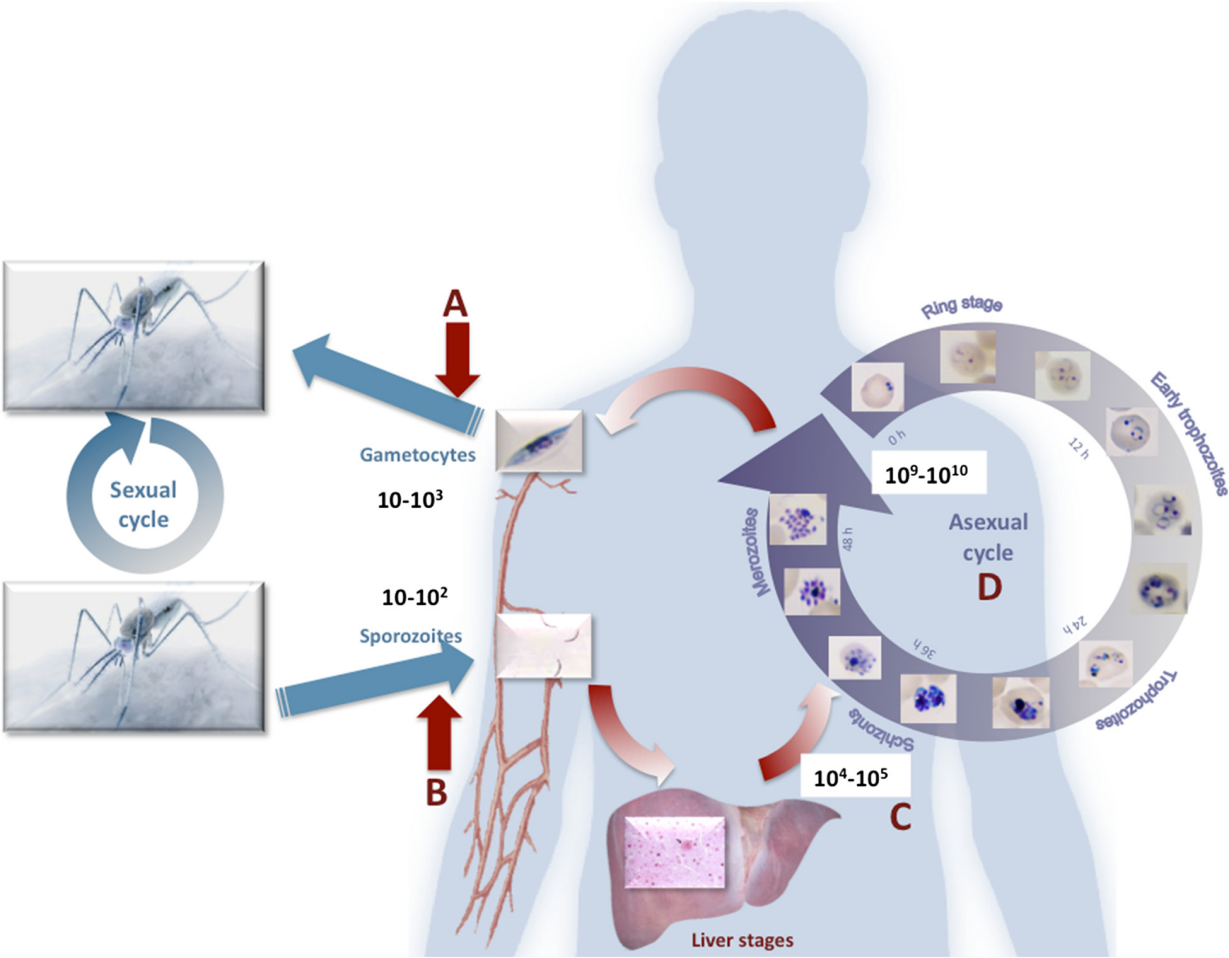
**Malaria parasite developmental cycles and possible targets for sustainable malaria control and elimination strategies.** Sporozoites are transmitted when female *Anopheles* mosquitoes take a human blood meal, where after the ~100 parasites transmitted infect human liver cells and mature for up to 14 days. Hundreds of thousands of daughter merozoites are subsequently released into the bloodstream to infect human erythrocytes and initiate the rapid and massive asexual replication cycle. Single parasites mature within 48 hours from rings to schizonts, releasing up to 32 daughter merozoites. Within a short amount of time, billions of parasites can be present in a patient’s bloodstream, resulting in the pathogenesis of the disease. Only a few parasites (<1,000) are required to develop into sexual gametocyte forms and allow transmission of the parasites back to the mosquitoes to undergo sexual replication in the insect vector. Targets within this complete developmental cycle that are viable in sustained control and elimination strategies include population bottlenecks (**A** and **B**) resulting in a block in transmission; targeting hypnozoite forms (*Plasmodium vivax*) or liver-stage maturation (**C**) and **D**, targeting the massive asexual replication cycle to treat patients symptomatic of the disease.

The parasite has an extremely complex life cycle, with sexual development in the mosquito vector and asexual replication in the human host’s erythrocytes (Figure 2). During the latter, the parasite is massively replicated reaching numbers in the billions. This makes effective targeting of this replication cycle almost impossible, as is evident from rapid evolvement of drug-resistant forms. Fortuitously, there are points in the parasite’s development where population bottlenecks exist, and this is particularly when the parasite resides in the mosquito vector or when 100 to 1,000 parasites are transmitted between humans and mosquitoes (Figure 2).

Various partnerships and consortia (e.g., Medicines for Malaria Venture [64,65]; CRIMALDDI [66]; and malERA [67]) emphasize the need for novel anti-malarials that are: 1) effective against erythrocytic stages and exo-erythrocytic stages of the parasite; 2) effective against resistant forms of the parasite; 3) chemically distinct and with new mechanisms of action; 4) safe without associated toxicities; 5) pharmacokinetically amenable to once-daily oral dosing; and 6) economically viable. Target product profiles for anti-malarials that meet these criteria and support the malaria-elimination strategy are have been identified and are broadly grouped into three areas: 1) control of the disease (treatment of infected patients); 2) blocking the transmission cycle; and 3) radical cures for malaria (e. g., targeting *P. vivax* hypnozoites in the livers of infected patients) [65,67].

Almost all currently used anti-malarials target various processes in the asexual, erythrocytic cycle with mostly unknown modes-of-action – including antibiotics (ciprofloxasin, doxycyclin, fosmidomycin, rifampicin), antifolates (pyrimethamine, proguanil and sulphadoxine), chloroquine and quinolone derivatives (amodiaquine, mefloquine), artemisinins and atovaquone. As the replication of the parasite in erythrocytes is responsible for malaria pathogenesis and death, parasite development in the erythrocytic cycle will always need to be one of the major focus areas for development of anti-malarial against novel targets to sustain malaria control strategies (Figure 2, Table 3). Therefore, research into new drugs and novel drug targets is still and will remain imperative and the useful lifetime of current anti-malarials (including artemisinin combination therapies) has to be maximized. Any new drug targeting the erythrocytic cycle will have to show a rate of killing comparative to its half-life [68] and at drug concentrations <10 nM *in vitro*. Pre-clinical candidates have to show good pharmacokinetic parameters including good oral bioavailability to be applicable in the field in malaria-endemic regions, especially also for children. Several new candidate drugs/drug targets are under investigation, including for example targeting dihydroorotate dehydrogenase [69] and the mitochondrial BC1 complex (atovaquone derivatives) [70]. High-throughput screening of libraries of ~4 million chemical entities (e.g. from Glaxo-SmithKlein, Novartis and St. Jude’s Childrens Hospital), have resulted in ~20 000 compounds with <1 μM activity against asexual-stage malaria parasites *in vitro*, which promises to be a invaluable resource in anti-malarial discovery programmes*.*

**Table 3:** **Potential sustainability of parasite control strategies in malaria control, elimination and eradication programmes**[64-67]

**Subgroup**	**Advantages**	**Disadvantages**	**Sustainability**
**Chemoprophylaxis: asexual stages**	Fast acting, prevents onset of infection and disease	Resistance developed	No, not if only targeting prevention of infection
**Chemoprophylaxis: transmission blocking**	Sporozoite stages and hepatocytic stages targeted, could have simultaneous prevention of onset of disease	Drug delivery and technical constraints	Yes
**Chemotherapy: treatment of disease**	Decrease parasite burden, treat malaria-associated symptoms	Resistance developed, new drugs and targets needed	Maybe, if drugs block erythrocytic development as well as formation of gametocytes
**Chemotherapy: antihypnozoite**	Treatment of P. vivax liver stage malaria	Technical constraints in drug development	Maybe, species specific eliminations
**Chemotherapy: transmission blocking**	Block human-mosquito transmission (gametocytocidal), could have simultaneous prevention of onset of disease	Technical constraints in drug development	Yes

Sustainable malaria control with chemotherapeuticals additionally requires targeting exo-erythrocytic and transmission-causing forms of the parasite (Figure 2, Table 3), especially dormant liver-stage infection with *P. vivax* hypnozoites, particularly in countries where *P. vivax* infection is rampant [71]. Only a single drug, primaquine, effectively targets hypnozoites and drug-discovery programs in this field are limited due to technical constraints in culturing liver-stage parasites of *P. vivax* and other *Plasmodium* species. Such an *in vitro* culturing ability will enable downstream developments of diagnostics methods and novel drugs. Several initiatives include 3D culture scaffolds for liver cell culturing [72] but require transdisciplinary approaches between material scientists, cell biologists and parasitologists.

One of the areas that could have the biggest impact in sustainable malaria control and malaria elimination is interruption of the transmission of the parasite between humans and mosquito vectors (Figure 2). The value of transmission-blocking drugs that target population bottlenecks in the parasite’s life cycle lies in the ability to target the parasite both in the host (gametocytes) as well as in the mosquito (sporozoites). Currently used but compromised anti-malarials, such as artemisinin and primaquine, already target some stages of gametocyte development, illustrating the potential of such an approach. Additionally, drugs targeting both asexual and sexual forms of the parasite would further enhance the value of such drugs [73]. However, gametocytocidal drugs could always be used in combination with drugs targeting asexual parasites, if comparative pharmacokinetic properties could be ensured. Novel methods for screening of compounds with gametocytocidal activity are available [74,75]. However, development of transmission blocking drugs requires transdisciplinary approaches of combining expertise from parasite biologists, medicinal chemists, human physiologists and entomologists with the advantage that the complete life cycle of the parasite from mosquitoes to human hosts [75] can be investigated to determine the efficacy of transmission-blocking interventions. This will allow early detection of unanticipated factors influencing a positive outcome – for example, parasite-resistance development, human host influences, etc.

One important constraints of the current anti-malarial arsenal is their limited chemical scaffolds, apparently similar modes-of-action and thus increased probability of developing shared mechanisms of resistance. Focused efforts on identifying chemically distinct compounds with proven novel modes-of-action is required to maintain a steady stream of new anti-malarials. Such an approach necessitates identification of parasite-specific processes that are clearly distinct from those of the human host and essential to parasite survival. Surprisingly little is known about basic biological processes governing the parasite’s pathogenesis. However, exploitation of the immense datasets resulting from functional genomics investigations of the parasite has not met the initial expectations, mostly due to a lack of understanding of specific processes in the parasite. This poses a major obstacle in devising new anti-malarial control tools for sustainable malaria control.

## Conclusions

Despite many of the challenges facing malaria control programmes, several countries have been able to achieve malaria elimination. Countries currently at malaria control and pre-elimination stages need to be vigilant and systematic in their pursuit of elimination to ensure sustainability. Continued funding by governments of malaria-endemic regions is imperative to further sustain and progress malaria control and elimination programmes. Moreover, the international community has to invest heavily in novel research and development agendas if the ultimate aim of malaria eradication is to be achieved.

**Figure 3:**
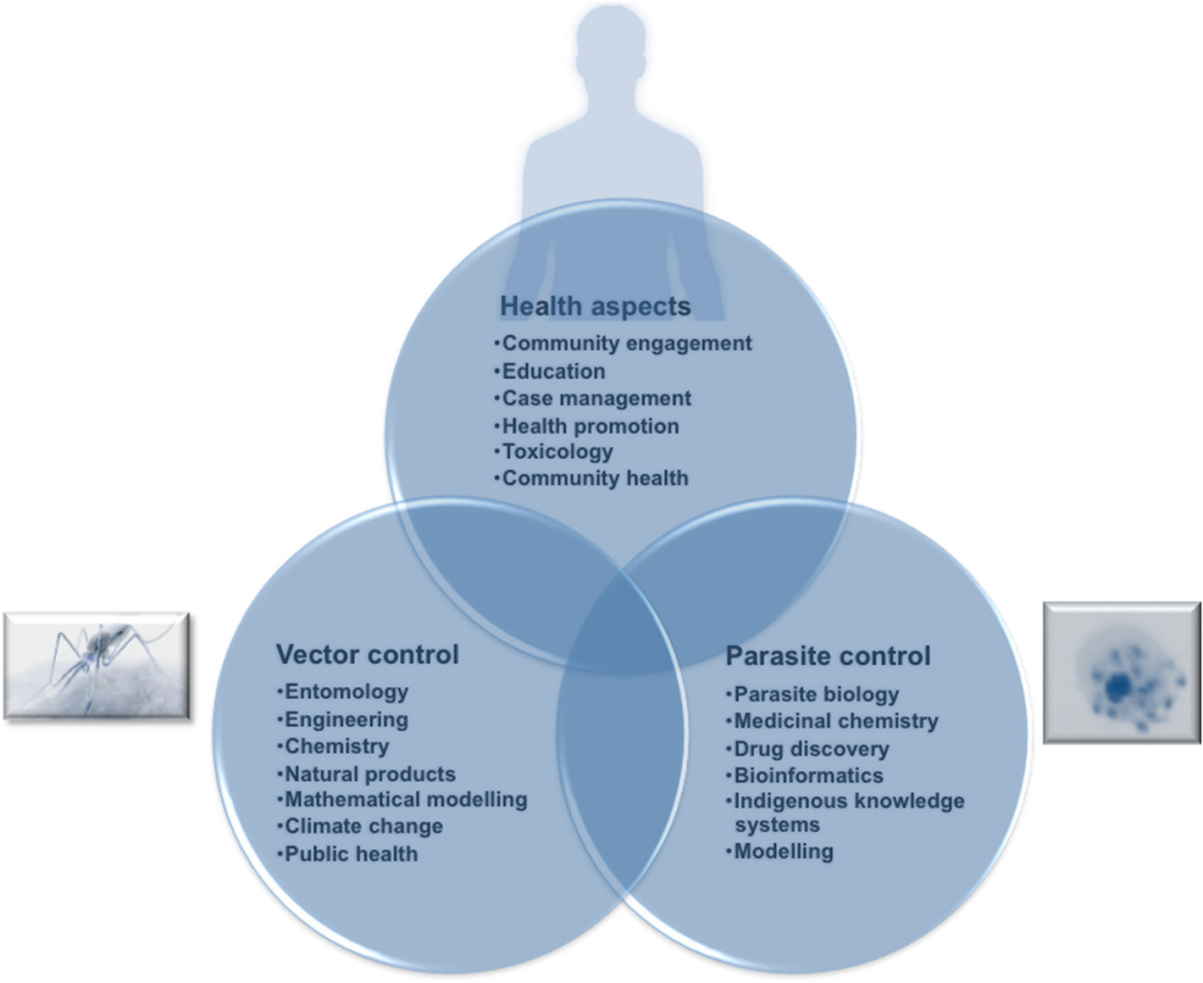
Transdisciplinary approaches to enable sustained malaria control and elimination, allowing translational applications to the malaria community.

Transdisciplinary approaches are essential in order to sustain control and elimination of this complex disease. Specific focus areas under the auspices of both vector and parasite control must include consideration of the impact of control measures on human health of the affected communities (Figure 3). In the context of parasite control, transdisciplinary initiatives combining expertise from parasite biology, medicinal chemistry, drug discovery, mathematical modelling and bioinformatics-based data mining and predictions, and indigenous knowledge systems provide opportunities to streamline the sustained pipeline of novel chemical entities that target weak points in the parasite’s biology. Vector control strategies are highly reliant on transdisciplinary approaches, as depicted by IVM. Thus, transdisciplinary measures include those that appear to contribute to six key elements of IVM: 1) integrated approaches requiring entomology, chemistry, biochemistry, engineering and environmental health; 2) inter-sectoral collaborations including health, agriculture, environment and planning sectors; 3) evidence-based decision making requiring entomologists, parasitologists, epidemiology, medicine and policy research; 4) Advocacy and social mobilization within social and political sciences and community health; 5) legislation within public health and environmental law and 6) capacity-building between e.g. social sciences and health education etc. Additionally, collaborations between vector biologists, entomologists, engineers, chemists, natural product specialists, mathematical modelling, climate change specialists and public health researchers would further strengthen such programmes. Community involvement, education and sustained malaria case management are essential for any application of transdisciplinary vector control programmes.

The successful integration of these transdisciplinary research approaches therefore holds immense promise for translationary applications, especially if they can originate in malaria-endemic countries in Africa that will lead to benefitting malaria-endemic communities and further ensures sustained malaria control and elimination.

## Abbreviations

DDT: Dichlorodiphenyltrichloroethane; DDE: Dichlorodiphenyldichloroethylene; IRS: Indoor residual spraying; ITN: Insecticide-treated net; ITWLs: Insecticide-treated wall linings; IVM: Integrated vector management; WHO: World Health Organization.

## Competing interests

The authors declare that they have no competing interests.

## Authors’ contributions

LMB conceived the idea for the manuscript, wrote the section on parasite control and overall manuscript preparation; RB: the section on adverse health aspects of vector control; WF: vector control strategies; CM: vector control strategies and integrated vector management; CdJ: overall manuscript preparation. All authors read and approved the final manuscript.
